# Research on Nonlinear Feature of Electrical Resistance of Acupuncture Points

**DOI:** 10.1155/2012/179657

**Published:** 2012-12-17

**Authors:** Jianzi Wei, Huijuan Mao, Yu Zhou, Lina Wang, Sheng Liu, Xueyong Shen

**Affiliations:** ^1^Laboratory of Acupuncture and Moxibustion, Shanghai University of Traditional Chinese Medicine, 1200 Cailun Road, Shanghai 201203, China; ^2^Systemic Physiology Laboratory for Acupuncture and Meridian, Shanghai Research Center of Acupuncture and Meridians, 199 Guoshoujing Road, Shanghai 201203, China

## Abstract

A highly sensitive volt-ampere characteristics detecting system was applied to measure the volt-ampere curves of nine acupuncture points, LU9, HT7, LI4, PC6, ST36, SP6, KI3, LR3, and SP3, and corresponding nonacupuncture points bilaterally from 42 healthy volunteers. Electric currents intensity was increased from 0 **μ**A to 20 **μ**A and then returned to 0 **μ**A again. The results showed that the volt-ampere curves of acupuncture points had nonlinear property and magnetic hysteresis-like feature. On all acupuncture point spots, the volt-ampere areas of the increasing phase were significantly larger than that of the decreasing phase (*P* < 0.01). The volt-ampere areas of ten acupuncture point spots were significantly smaller than those of the corresponding nonacupuncture point spots when intensity was increase (*P* < 0.05 ~ *P* < 0.001). And when intensity was decrease, eleven acupuncture point spots showed the same property as above (*P* < 0.05 ~ *P* < 0.001), while two acupuncture point spots showed opposite phenomenon in which the areas of two acupuncture point spots were larger than those of the corresponding nonacupuncture point spots (*P* < 0.05 ~ *P* < 0.01). These results show that the phenomenon of low skin resistance does not exist to all acupuncture points.

## 1. Introduction

Skin resistance, one of the important biophysical indexes supporting the objective reality of acupuncture points, had been detected since the 1950s, when Voll [1] found that skin points electrical characteristics in traditional acupuncture points areas were different from other areas. Since then, a number of studies have concluded that acupuncture points exhibit low skin resistance [2–4] and increased conductivity [5–7], but different opinions on the electrical characteristics of acupuncture points have remained. Pei and Liu put forward their dissent years ago [8], while Korr et al. found that the distribution of low-resistance areas varied from individual to individual, and not all were located at acupuncture points [9]. Recently, Pearson et al. reported that right GB 14, right PC 8, and left TE1 showed no low skin resistance at all [10]. Kramer et al. tested six commonly used acupuncture points on 53 subjects and found that the occurrence rate of low resistance was only 25.9 percent [11]. Besides the complexity of detecting skin resistance at acupuncture points, which may be influenced by numerous factors [12, 13], the root of these contradictory results is that skin resistance of acupuncture points is nonlinear. Because of the complexity of its composition, acupuncture points electrical impedance has some typical characteristics different from linear impedance component (e.g., the space distribution of acupuncture point tissues is anisotropic, and the acupuncture point tissues are actively responsive to external input). Thus, acupuncture point electrical impedance is nonlinear and flexible; its values change when the detected voltages or currents vary [14]. Since the resistance value of an acupuncture point varies according to current and voltage, the electrical characteristics of acupuncture points must be expressed by a volt-ampere curve.

Classification of the electrical properties of acupuncture points will provide an objective index for studying the functions of the points. Active response to external stimulation and the reflection of symptoms are the main characteristics of acupuncture points, of which the former is more important as it is the means by which acupuncture can be applied therapeutically. When external stimuli such as needling, moxibustion, electrostimulation, lasers, and infrared irradiation act on acupuncture points, they may produce obvious reactions that subsequently regulate and balance bodily functions. However, due to the lack of objective indexes, research on the active response of acupuncture points to external stimulation is still quite limited.

The purpose of this study is threefold: to use a nonlinear detecting method to ascertain the volt-ampere curves of acupuncture points and observe their basic features, to compare volt-ampere areas of acupuncture points and non-acupuncture points to determine their skin resistance, and to probe the relationship between an acupuncture points' volt-ampere characteristics and its response to external stimulation.

## 2. Methods

### 2.1. Subjects

42 healthy volunteers, 23 males and 19 females, aged 23–36, with a mean age of 26.73 ± 3.02, were recruited. “Healthy” was defined as normal body temperature and no known autonomic nervous system dysfunction, coronary heart disease, systemic disease, or skin disease [15]. All subjects were informed of the nature of the experiment and willingly signed the consent form before participation. The research protocol was approved by the Human Study Ethics Committee of the Shanghai Research Center of Acupuncture and Meridians.

The acupuncture points tested were bilateral LU9 (Taiyuan), HT7 (Shenmen), LI4 (Hegu), PC6 (Neiguan), ST36 (Zusanli), SP6 (Sanyinjiao), KI3 (Taixi), LR3 (Taichong), SP3 (Taibai) and; thus, 18 acupuncture point spots and their relevant non-acupuncture points controls. The control for LU9 was the midpoint between LU9 and PC7 (Daling). The control for HT7 was the midpoint between HT7 and PC7. The control for LR3 and SP3 was the midpoint between these two acupuncture points. Each control for the remaining acupuncture points was on the same level as the respective point and 1 cm lateral to it.

### 2.2. Experimental Device

**Figure 1:**
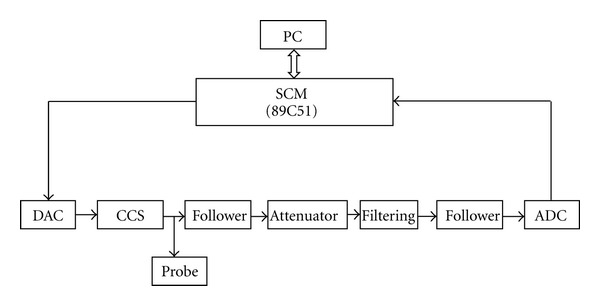
Hardware schematic diagram of volt-ampere characteristic detection system. A highly sensitive volt-ampere characteristic detecting system was constructed using a single chip micyoco (SCM), a digital-analog component (DAC), a constant current source component (CCS), a voltage follower, an attenuator, an analog-digital component (ADC), and two electrodes. The detecting electrode was a plane electrode installed a silver head 4 mm in diameter. The negative electrode was silver-gilt like that of the limb lead of an electrocardiograph. The SCM was connected to the USB of a personal computer (PC) through a data line.

A highly sensitive volt-ampere characteristic detecting system and its working mechanism are used as referenced in [16, 17] (see Figure 1).

### 2.3. Experimental Procedures

The experiment was performed under quiet, controlled environmental conditions: temperature 22°C ± 3°C, minimal air flow, relative humidity 55 ± 10%, and shielding from electromagnetic radiation [17].

The subjects were asked to arrive at the laboratory more than 15 minutes prior to the experiment and sit quietly to relax their muscles to become acclimated to the testing conditions. The detecting electrode was placed on the points with 160 g ± 5% pressure ten minutes after 75% alcohol was applied to the detected spots.

The negative electrode was applied with cardio cream made by Nihon Kohden Corporation. When LU9, HT7, PC6, and their relevant control points were detected, the inert electrode was placed three cun from the cubital crease on the palm aspect of the forearm. When ST36, KI3, SP3, LR3, and their relevant control points were detected, it was placed three cun above the tip of the medial malleolus. When SP6 and its control point were detected, it was placed three cun below ST35 (Dubi).

**Figure 2:**
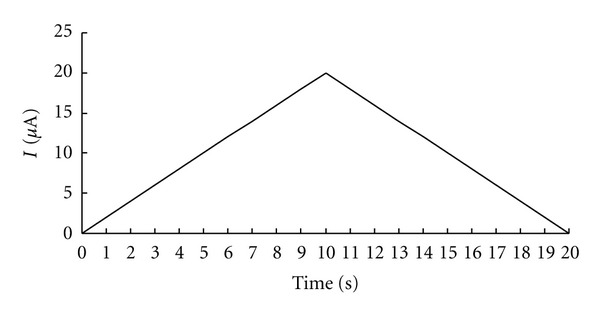
Electrical current-time *I*-*T* curve of detecting system. The electrical current first increase from 0 *μ*A to 20 *μ*A (0–10 s), than decrease from 20 *μ*A to 0 *μ*A (10–20 s).

The parameters of the detecting system were adjusted to 30V, the maximum voltage detection range, and 0 *μ*A–20 *μ*A, the scanning range of constant current, in order to avoid stimulating or polarizing effects [18]. The total scanning time was 20 seconds. The electrical current scanning was increasing steadily and lineally from 0 *μ*A to 20 *μ*A in the first 10 seconds and decreasing steadily from 20 *μ*A to 0 *μ*A in the second 10 seconds. The data was recorded every 0.1 *μ*A; each spot was designed to measure 3 times in advance, and the value showed was already the average of them (see Figure 2).

**Figure 3:**
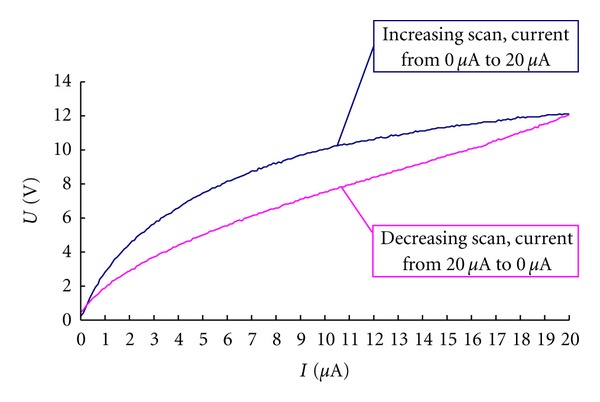
Schematic diagram of volt-ampere curve of the acupuncture points.

The system recorded the value of the corresponding voltage automatically, and during the biphase scanning, it traced the volt-ampere curve as well. Each detected spot had two volt-ampere curves, recorded respectively with 0 *μ*A~20 *μ*A and 20 *μ*A~0 *μ*A (see Figure 3).

### 2.4. Statistics

The original data was filtered before statistical analysis. If the voltage at the acupuncture points or control points of a subject exceeded 30V, that whole data wasn't fit into statistics.

The volt-ampere area refers to the area comprised by the volt-ampere curve and abscissa, which was calculated by integral calculus (∫UdI). As the data were collected discontinuously, the volt-ampere area was calculated as *S* = Σ*U*Δ*I*.

The SPSS10.0 software package was used, and the paired-sample *t*-test was applied to compare the difference of volt-ampere areas between acupuncture points and control points as well as the difference between the volt-ampere areas of the increasing and decreasing scans of each point. In all cases, *α* = 0.05 or less was considered significant.

## 3. Result

Bilaterally, thirty-four spots were scanned on 42 healthy volunteers: nine bilateral acupuncture points and eight relevant bilateral control points, since LR3 and SP3 shared the same control.

**Figure 4:**
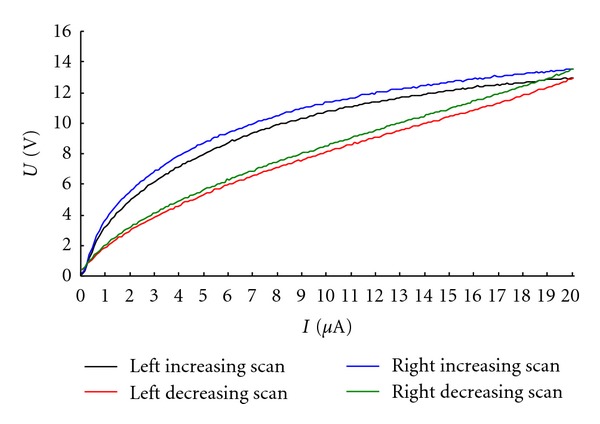
Volt-ampere curve of PC 6. The volt-ampere curves of acupuncture points were parabola-like rather than straight. The curves produced by increasing current intensity did not totally overlap those produced by decreasing current intensity.

Qualitative analysis showed that all detected spots had nonlinear and magnetic hysteresis-like characteristics. With regard to nonlinearity, the volt-ampere curves of acupuncture points were parabola-like rather than straight. This was most obvious at the very weak current, 0 *μ*A to 5 *μ*A, in the course of scanning from 0 *μ*A to 20 *μ*A. During decreasing scanning, that is from 20 *μ*A to 0 *μ*A, they became almost straight (see Figure 4). With regard to magnetic hysteresis, the curves produced by the increasing intensity did not totally overlap those produced by decreasing intensity. This was quite similar to the B-H curves that occur in ferromagnetic materials (see Figure 4).

Further quantitative analysis showed that in both acupuncture points and the control the volt-ampere areas produced by the increasing intensity were all larger than those of the decreasing intensity (*P* < 0.01) (see Table 1).

When the acupuncture points and control points were compared, four acupuncture point spots, bilateral LI4 and bilateral SP3, had larger volt-ampere areas than those of their relevant control spots when intensity was increasing, but the differences were not statistically significant. The other fourteen acupuncture point spots had smaller volt-ampere areas than their relevant control spots did. The differences in ten of these, five bilateral points, reached statistical significance (*P* < 0.05 ~ *P* < 0.001). These were LU9, PC6, ST36, SP6, and KI3 (see Table 1).

During decreasing intensity, five acupuncture point spots, right HT7, bilateral ILI4, and bilateral SP3, had larger volt-ampere areas than their relevant control spots did; the differences for right LI4 and right SP3 reached statistical significance. Each of the other thirteen acupuncture point spots had smaller volt-ampere areas than those of their relevant control spots; of these, eleven acupuncture point spots reached statistical significance (*P* < 0.05 ~ *P* < 0.001). They were bilateral LU9, bilateral PC6, bilateral ST36, bilateral KI3, bilateral LR3, and left SP6 (see Table 1).

## 4. Discussion

Our data showed that the volt-ampere curves of acupuncture points are clearly nonlinear (Figure 3), further substantiating previous reports [19–21] and indicating that only with nonlinear detecting methods can the panorama of electrical characteristics of acupuncture points be obtained. Conversely, linear detecting methods will not yield precise results even when experimental conditions are strictly controlled.

Besides their methodological significance, the nonlinear characteristics of the volt-ampere curve also have biological significance. The human being is both a complex living organism and a complicated open system. The behaviors and physiological activities of the human body possess the features of nonlinearity and unpredictability that correspond to complex systems. During the course of evolution, the human body has adapted to its environment. Homeostasis regulates and maintains the cells to play their physiological roles smoothly, permitting the body to eliminate various kinds of internal and external factors that disturb the normal physiochemical state of its internal environment. As a result, a dynamic balance of the internal environment can be maintained. The nonlinearity of skin resistance, one of the biophysical characteristics of acupuncture points, may be regarded as a response to the complexity of the functions of the body.

The volt-ampere area is the integral of voltage response resulting from the scanning current on the acupuncture points. It is the average strength of the current during the time of scanning, and its value reflects the resistance/impedance of the acupuncture points to that scanning current. A higher value at the volt-ampere area means greater resistance/impedance.

In this study, left and right LI4 and left and right SP3 had larger volt-ampere areas than the relevant control during the increase, as did the right HT7, left and right LI4, and left and right SP3 during the decrease, among which right LI4 and right SP3 reached statistical significance. Thus, not all acupuncture point spots volt-ampere areas were smaller than those of control. This indicates that the phenomenon of low acupuncture points skin resistance does not always exist, which is consistent with the results of our previous research [17, 18, 20]. The data showed that acupuncture points skin resistance may either be lower or higher, depending on different points. The prevalent methods of detecting acupuncture points according to the lower skin resistance are not reliable.

Acupuncture points actively respond to external electric stimulation. In this study, two manifestations were observed when acupuncture points were stimulated by increasing and decreasing electric current intensity. First, the degree of nonlinearity declined as the scanning time went on, and second, volt-ampere curves from 0 *μ*A to 20 *μ*A were totally different from those of 20 *μ*A to 0 *μ*A (see Figure 2). When analyzed quantitatively, the volt-ampere areas during the decreasing intensity were smaller than those of the increasing intensity (Table 1). The active response to external electric stimulation further confirms that an acupuncture point is sensitive to stimulation, as do the myoelectric signal sent out when an acupuncture point is needled [22] and the degranulation of a mastocyte when an acupuncture point is stimulated by needles [23] or lasers [24].

As in our previous research [21, 25–27], the data suggests that the volt-ampere areas under increasing intensity and those under decreasing intensity vary with physiological and pathological changes of the body. Does this suggest that acupuncture points respond differently to external stimulation under different physiological and pathological states? Can the difference values be regarded as an index for studying acupuncture points response to external stimulation? This warrants further study.

The volt-ampere curve of the increasing intensity was totally different from that of the decreasing. The fact that it was larger when intensity was increasing suggests a similarity to the magnetic hysteresis characteristics of ferromagnetic materials. The magnetic hysteresis-like characteristic of the volt-ampere curve of an acupuncture point may be quantitatively expressed by the difference value, that is, the value of the volt-ampere area increasing scanning minus that of decreasing scanning. With Potter's method, Chen studied the resistance characteristics of the heart meridian on the forearm. It was found that an acupuncture point has the general properties of a resistance-capacitor circuit. Topologically it was the parallel connection of a resistance and a capacitor, which has a series connection with a resistance [28]. The same results were obtained when Reichmanis applied Laplace analysis to the segment of the heart meridian from HT4 to HT3 [3]. Since acupuncture points possess resistance-capacitor circuit-like properties which can store and release electric current, this may explain the magnetic hysteresis-like phenomenon of the volt-ampere curves of acupuncture points in this study. The time curves of the electric current under increasing and decreasing intensity were symmetrical (see Figure 2), but the volt-ampere area during the increase was larger. This demonstrates that some part of the current going into the acupuncture point during the increase must have remained in the acupuncture point, suggesting that acupuncture points have the ability to store energy and also warrants further study.

## 5. Conclusion

The phenomenon of low skin resistance does not exist at all acupuncture points. The volt-ampere curves of human acupuncture points are clearly nonlinear; consequently, nonlinear detecting methods should be applied. The volt-ampere curve-detecting device used in this study is noninvasive and convenient and provides a valuable new method for studying acupuncture points response to external stimulation. Although only the volt-ampere characteristics were detected, the complete picture of electrical characteristics can be obtained with the device. The magnetic hysteresis-like characteristics of the volt-ampere curve of acupuncture points indicate that the points store part of the electric energy they get during external electrical stimulation. Our main focus during followup will concern the relationship between the energy of acupuncture points and their functions.

## Figures and Tables

**Table 1:** Volt-ampere areas of acupuncture points at human bodies.

		Right increasing scan	Left increasing scan	Right decreasing scan	Left decreasing scan
LU9	(*n* = 39)	169.83 ± 104.30^∗∗∗∗∧^	172.82 ± 89.55^∗∗∗∗∧^	151.95 ± 77.63****	154.36 ± 66.58****
Control point of LU9		249.27 ± 97.82^∧^	227.31 ± 103.34^∧^	218.89 ± 76.96	198.43 ± 74.39
HT7	(*n* = 37)	272.30 ± 113.63^∧^	245.28 ± 109.48^∧^	239.73 ± 88.99	217.69 ± 87.27
Control point of HT7		277.38 ± 103.41^∧^	255.53 ± 94.83^∧^	238.10 ± 79.92	226.31 ± 70.41
LI4	(*n* = 36)	244.83 ± 106.56^∧^	244.40 ± 115.35^∧^	197.87 ± 71.71*	191.88 ± 77.26
Control point of LI4		226.53 ± 102.00^∧^	227.89 ± 111.78^∧^	178.56 ± 70.65	182.05 ± 76.23
PC6	(*n* = 38)	208.72 ± 112.58^∗∗∧^	190.58 ± 92.47^∗∗∧∗^	164.05 ± 79.60***	151.38 ± 60.60****
Control point of PC6		231.81 ± 124.30^∧^	213.01 ± 97.60^∧^	183.09 ± 87.69	166.43 ± 64.45
ST36	(*n* = 34)	178.14 ± 89.23^∗∗∗∗∧^	186.57 ± 109.47^∗∗∗∗∧^	141.57 ± 61.71****	147.46 ± 72.01****
Control point of ST36		221.76 ± 98.97^∧^	240.41 ± 124.20^∧^	172.61 ± 65.90	179.18 ± 82.20
SP6	(*n* = 35)	225.24 ± 104.14^∗∗∗∧^	231.55 ± 106.51^∗∧^	178.29 ± 75.88	179.91 ± 71.66**
Control point of SP6		258.02 ± 122.97^∧^	252.07 ± 114.07^∧^	188.38 ± 81.09	193.26 ± 73.30
KI3	(*n* = 28)	187.65 ± 116.88^∗∗∗∗∧^	209.78 ± 114.49^∗∧^	157.43 ± 81.57****	169.14 ± 79.60**
Control point of KI3		257.00 ± 146.53^∧^	262.43 ± 134.84^∧^	213.14 ± 112.28	212.54 ± 90.84
LR3	(*n* = 34)	212.60 ± 100.70^∧^	220.67 ± 103.65^∧^	177.58 ± 69.11**	180.98 ± 68.45***
Control point of LR3 and SP3		227.19 ± 104.57^∧^	245.27 ± 95.29^∧^	197.35 ± 73.69	211.21 ± 65.99
SP3		247.47 ± 100.95^∧^	252.68 ± 90.14^∧^	229.31 ± 82.53***	228.73 ± 70.15

Compare to decreasing-scan ^*∧*^ *P* < 0.01.

Compare to control point *P < 0.05; **P < 0.01; ***P < 0.005; ****P < 0.0001.
